# Attention-enhanced architecture for improved pneumonia detection in chest X-ray images

**DOI:** 10.1186/s12880-023-01177-1

**Published:** 2024-01-02

**Authors:** Dikai Li

**Affiliations:** Shenzhen Key Laboratory of Ultraintense Laser and Advanced Material Technology, Center for Advanced Material Diagnostic Technology, and College of Engineering Physics, Shenzhen Technology University, Lantian Road, Shenzhen, Guangdong 518118 China

**Keywords:** Pneumonia detection, Attention-enhanced architecture, Imbalanced training samples, Medical imaging

## Abstract

In this paper, we propose an attention-enhanced architecture for improved pneumonia detection in chest X-ray images. A unique attention mechanism is integrated with ResNet to highlight salient features crucial for pneumonia detection. Rigorous evaluation demonstrates that our attention mechanism significantly enhances pneumonia detection accuracy, achieving a satisfactory result of 96% accuracy. To address the issue of imbalanced training samples, we integrate an enhanced focal loss into our architecture. This approach assigns higher weights to minority classes during training, effectively mitigating data imbalance. Our model’s performance significantly improves, surpassing that of traditional approaches such as the pretrained ResNet-50 model. Our attention-enhanced architecture thus presents a powerful solution for pneumonia detection in chest X-ray images, achieving an accuracy of 98%. By integrating enhanced focal loss, our approach effectively addresses imbalanced training sample. Comparative analysis underscores the positive impact of our model’s spatial and channel attention modules. Overall, our study advances pneumonia detection in medical imaging and underscores the potential of attention-enhanced architectures for improved diagnostic accuracy and patient outcomes. Our findings offer valuable insights into image diagnosis and pneumonia prevention, contributing to future research in medical imaging and machine learning.

## Introduction

In the pursuit of assessing the widespread applicability of our artificial intelligence system, we employed this transfer learning framework to the diagnosis of pediatric pneumonia, a prevalent and devastating disease. As per the data from the World Health Organization (WHO), pneumonia is responsible for approximately two million annual deaths in children under the age of 5, thereby emerging as the foremost cause of child mortality [1], surpassing the combined total of deaths from HIV/AIDS, malaria, and measles [2]. The WHO report indicates that 95% of the newly diagnosed pediatric clinical pneumonia cases primarily occur in developing countries, particularly in Southeast Asia and Africa. The etiological agents of pneumonia predominantly are bacteria and viruses, each, requiring different treatment strategies. Bacterial pneumonia necessitates an immediate referral for prompt antibiotic therapy, while viral pneumonia is managed with supportive care. As such, a precise and timely diagnosis is of paramount importance. A critical factor in diagnosis is radiological data, as chest X-rays routinely acquired as as standard care and can aid in differentiating various types of pneumonia. However, rapid interpretation of radiographic images often proves infeasible, especially in resource-constrained settings where the incidence and mortality of pediatric pneumonia are highest. To address this, we also investigated the effectiveness of our transfer learning framework in classifying pediatric chest X-rays to detect pneumonia and further discern between viral and bacterial pneumonia, with the aim of facilitating rapid referrals for children in need of urgent interventions.

### Medical imaging differences

Pneumonia is a lung infection instigated by bacteria, viruses, or fungi. This infection leads to inflammation in the alveoli and fluid-filled areas in the lungs (pleural effusion). As a major cause of infant mortality and global deaths, pneumonia’s prevalence is particularly potent in overcrowded, polluted, and unsanitary environments prevalent in underdeveloped and developing countries. These conditions, coupled with a lack of medical resources, precipitate pneumonia outbreaks. Our work aims to provide a promising approach for accurately and efficiently detecting pneumonia from chest X-ray images, benefiting millions of people globally. Early detection and treatment are crucial to prevent fatal conditions. X-rays, CT scans, MRI, are employed to diagnose pulmonary diseases, with X-rays being the most commonly used method for diagnosing pneumonia. The proposed architecture will assist radiologists in accurately analyzing these images, potentially facilitating the diagnosis of other respiratory diseases, fractures, and tumors.

Characteristic alterations can be observed in X-ray images between patients with pneumonia and healthy individuals. Although these differences may be subtle, they hold significant importance in medical diagnosis. The following are some potential differences that may exist: 1. Infiltration areas: X-ray images of pneumonia patients typically exhibit various degrees of infiltration in the lungs, which appear as hazy or blurry shadows in the images. These infiltrated areas may result from inflammation-induced lung tissue damage, fluid accumulation, or pathogen infections. 2. Spots or nodules: X-ray images of pneumonia patients may show spots or nodules, which present as small circular or elliptical lesions. These spots or nodules are often associated with infection or inflammation and may serve as indicators of pulmonary lesions. 3. Air-space consolidation or shadows: In X-ray images of pneumonia patients, air-space consolidation or shadows may be observed, indicating that the alveoli in the lungs are filled with fluid or exudate. This condition is likely caused by an inflammatory response due to lung infection. 4. Changes in lung density: X-ray images of pneumonia patients might reveal alterations in lung density, which could be a result of lung tissue being affected by infection or inflammation. Density changes may include lung tissue infiltration, condensation, or diffusion. 5. Pleural thickening: X-ray images of pneumonia patients may reveal pleural thickening, which refers to an increase in thickness of the pleura lining the lungs within the chest cavity. Pleural thickening is often associated with lung infection or inflammation.

These distinctions observed in X-ray images provide important diagnostic information and aid in the distinction between pneumonia patients and healthy individuals.

It is important to note that these differences may be relative, and the characteristic manifestations of pneumonia may vary depending on individual differences. For accurate diagnosis and assessment, medical professionals typically consider multiple aspects of the X-ray, such as the location, shape, and size of infiltrated areas. Concurrently, they also take into account the patient’s symptoms and other clinical information for comprehensive evaluation. Therefore, the diagnosis and judgment by trained medical professionals are crucial for confirming pneumonia.

### Our work

**Fig. 1:**
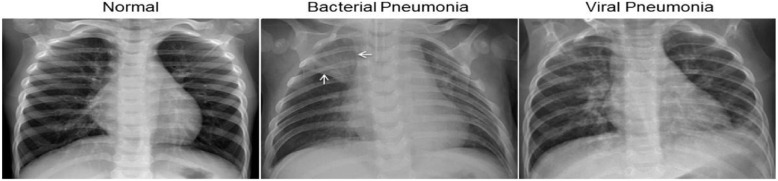
Comparison of normal lung X-ray and pneumonia X-ray photos

One of the key strengths of our work lies in its ability to effectively capture complex spatial and channel correlations in chest X-ray images, which is crucial for accurate pneumonia detection. Figure 1 illustrates a chest X-ray image of a person with pneumonia and a healthy individual. The white spots on the right X-ray image indicate the presence of pneumonia. Due to the potential subjectivity influenced by radiologists’ experience, pneumonia detection from X-rays underscores the necessity for computer-aided detection and diagnosis to obtain accurate results. Deep neural networks have demonstrated remarkable potential in image classification tasks. In our research, we constructed a Convolutional Neural Network (CNN) [3], an extension of traditional neural networks, capable of extracting the most representative features from multi-dimensional input objects. This is achieved by correctly encoding the spatial locations and orientations of image channels in the input images.

In recent years, attention mechanisms have gained significant interest in computer vision systems, used for object recognition and scene interpretation. Mirroring the human visual system’s capability to rapidly distinguish objects by focusing only on their relevant parts, the concept of integrating attention mechanisms into deep neural networks emerged. Attention mechanisms have seen wider application in activities related to natural language processing. Recently, they have also been used in image classification tasks, yielding state-of-the-art outcomes. Channel attention and spatial attention are two typical attention mechanisms applied in computer vision tasks.

Recently, efforts have been made by researchers to augment the Convolutional Neural Network by incorporating channel and attention modules from residual networks to enhance the performance of pneumonia prediction from chest X-ray images. The innovation in this work lies in the addition of an attention branch between layers of the convolutional neural network. This enables the network to focus on learning important regions from chest X-ray images while attending to complex spatial features, thereby boosting the precision of pneumonia detection. Our analysis of feature maps and attention maps demonstrates that the proposed model can effectively learn features from critical regions in chest X-ray images, thereby enhancing the performance of pneumonia detection using the proposed framework.

In this experiment, our main contributions are as follows:

1. We initially developed a unique attention mechanism built upon the residual neural network and evaluated its performance in detecting pneumonia using chest X-ray image datasets.
2. Following this, we integrated an enhanced focal loss into the aforementioned architecture, thereby substantially improving the model’s performance in scenarios with imbalanced training samples. We evaluated the effectiveness of this attention-enhanced architecture.
3. Moreover, we conducted a comparative analysis of the two architectures’ performance in order to gauge the impact of the incorporation of spatial and channel attention modules.

## Literature review

In this context, we provide the necessary background concepts for the proposed design and conducted a comparative study of recent research findings related to the same problem domain.

### Attention mechanisms

In image processing involve the identification of target regions, akin to how our eyes rapidly scan an image. When smaller activation values are integrated through relevant feature maps, a significant amount of information may be discarded; therefore, incorporating both spatial and channel attention in the quaternion residual network can yield better results. Moreover, attention mechanisms [4] prioritize regions of interest instead of feature maps. While channel attention reduces information from individual feature maps, spatial attention emphasizes many critical areas within each feature map by utilizing attention masks from different branches. In the final stage, the output feature maps from both attention processes are concatenated. These regions of interest are amplified in the fused feature map, while redundant features are discarded. To collect the most accurate target data while reducing unnecessary data, the target regions are weighted (distributed). Soft attention [5], which is differentiable and permits end-to-end training of CNN models. Most soft attention models use attention templates to locate unique aspects that align weights for discrete sequences or image segments. In contrast to soft attention, hard attention, a stochastic and non-differentiable process, focuses on the the primary features of an image rather than on various regions.

Attention networks utilized for image classification can compute the arithmetic mean weight of the image attention spectrum. This method can collect image-based attention in a manner similar to techniques used in natural language processing.

Deep neural networks can perform pixel-level classification on images as they can extract features from data. Attention mechanisms mimic human vision and aid in rapid and accurate recognition of critical features. CNNs process all information and details of an image in all convolutional layers. Multiple convolutional layers and the final global average pooling average the features and attributes of an image. The last fully connected layer of the network determines the image’s classification. As the image size decreases, the impact of background and other non-critical information on the classification results becomes more significant. A large amount of data along with a neural network that does not emit background information can prevent inaccurate results.

One approach to generate an image from two or more convolutional layers is to use the output of branching output layers. We set the activation function sigmoid of the convolutional output is set to a sigmoid function, ensuring that the values for each pixel fall within the range of 0 to 1. The sigmoid function serves to constrain input values within this range. The result of the convolution function is multiplied with the initial output. Subsequently, two additional layers evaluate the output’s quantity. Values close to zero are considered unimportant, thereby leading to the dismissal of most sigmoid values close to zero, reducing the downstream recognition process. The configuration of the neural network to use the result to estimate the focus region is the most common method of applying attention mechanisms in image clasification tasks.

The existing literature presents two attention strategies inspired by the human visual system. The first is a top-down approach that iteratively selects the correct region from the scene record pool. However, the bottom-up approach highlights the most critical visual pathway locations. The top-down iteration is slower than the bottom-up method. Bottom-up techniques progressively select the most relevant regions from the incoming data, although sequential processing increases the depth of errors.

Classification neural networks model data as numerical vectors with equal weights for low-level features. The attention mechanism enables a model to selectively weigh different parts of the input sequence based on their relevance to the current prediction. It introduces a context vector that captures the importance of each input element, and this vector is used to compute a weighted sum of the input elements to produce the final output. By doing so, the model can emphasize the most informative parts of the input and ignore irrelevant or less important elements, leading to improved performance in various sequence modeling tasks.

$$\begin{aligned} \text {Attention}(Q, K, V) = \text {softmax}\left( \frac{QK^{T}}{\sqrt{d_{k}}}\right) V \end{aligned}$$

Where:

*Q*, *K* and *V* represent the query, key and value matrices. They are obtained by linear projections of the input sequence X using the weight matrices $W_Q$, $W_K$, and $W_V$. The symbol $d_k$ denotes the dimension of the key and query vectors. This is typically set to the square root of the dimension of the input embeddings or hidden states, in order to stabilize the attention scores.According to the above equations, the vector ${QK^T}$ provides important information to the vector *V*.

Attention mechanisms are a highly researched topic for several reasons. Foremost, any model with an attention mechanism outperforms baseline techniques. Additionally, attention models can be trained using basic recurrent neural networks through back-propagation. The advent of the Transformer model has been widely applied in image processing, video processing, and recommendation systems, improving attention models and avoiding parallelism issues in recurrent neural networks.

In our proposed work, we combine channel attention and spatial attention mechanisms in a residual network with side connections. A channel attention map is created, utilizing the relationships between features. Treating each channel of the feature map as a feature detector, channel attention focuses on global features. It reduces the spatial dimension of the input feature map. The channel attention map is then generated, which leverages the relationships between these features. The channel attention methodology yields a one-dimensional tensor for a designated feature map, emplying sigmoid activation. For some channel axes of the feature map, some activation values of the one-dimensional tensor to be larger than those of the corresponding interested feature map, while other activation values are smaller to avoid duplications of the feature map.

### Supervised pretraining

Typically, supervised learning involves training a model for classification using large-scale natural image datasets, such as ImageNet [6], to facilitate transfer learning. Research has demonstrated that using large-scale generic supervised pretraining models can yield numerous benefits, including accelerated training and improved performance in downstream tasks. Big Transfer (BiT) enhances this process by incorporating minor architectural modifications [7] and more recent training procedures, resulting in superior transfer learning performance and achieving state-of-the-art outcomes in various downstream transfer learning tasks. To harness these benefits, we initialize the backbone encoder using the weights obtained from minimizing the supervised cross-entropy loss in the BiT model trained on the JFT dataset. Given that deployment settings often limit the size of usable machine learning models (in terms of the number of model parameters), our approach is pivotal as it effectively accommodates both small and large model architecture sizes. To investigate this in more depth, we select two commonly used ResNet [8] architectures with varying depth and width multipliers: ResNet-50(1$\times$) and ResNet-152(2$\times$) as the backbone encoder networks. The pretrained encoder network derived from this stage undergoes denoted as $f_\phi (\cdot )$, and it undergoes further fine-tuning with medical domain data in subsequent pretraining steps.

### Comparative analysis of recent relevant research

The issue of detecting pneumonia through chest X-rays has persisted unresolved for many years, with a major mainly due to the lack of publicly available data.Traditional machine learning algorithms, which necessitate domain expertise for feature extraction, have been the focus of extensive research. With the advent of deep learning models, a variety of architectures have been introduced, including VGGNet [9], ResNet [8], and Inception ResNet.These architectures leverage pre-trained weights for transfer learning techniques. Recent strategies for pneumonia detection can be divided into three categories: (1) methods prioritizing the extraction of regions of interest, (2) approaches that emphasize feature extraction, followed by the use of typical machine learning models or model ensembles for performance averaging, and (3) deep learning architectures based on transfer learning.

## Dataset description

Currently,the ChestX-ray14 dataset [10], published by Wang et al. (2017), is one of the most commonly used for pneumonia classification. The dataset comprises 112,120 frontal chest X-ray images from 30,085 patients. Each X-ray image in the dataset is annotated with one or more of the 14 different thoracic diseases.

For our study, we constructed a new dataset, building upon the previous work. We sourced our dataset from a collection of X-ray images pertaining to 5,856 pediatric patients aged 1 to 5 years. These patients had visited the Guangzhou Women and Children’s Medical Center. The dataset constrains two types of X-ray images (in JPEG format): those showing pneumonia and normal chest X-rays (anterior-posterior views). All these images were part of the routine clinical care provided to the patients.

During the initial data analysis phase, we meticulously assessed the quality of the chest X-ray images. We excluded any low quality or those that were unreadable to maintain the integrity of our data samples. Following this, two expert physicians independently evaluated the X-ray images, providing diagnoses based on their observations.These assessments were critial to the subsequent the training phase of our artificial intelligence system. To account for any potential discrepancies or errors in these evaluations, we had a third expert review the assessment set. Harness final curated dataset was divided into two subsets: the training set and the validation set, each containing both types of X-ray images: normal and pneumonia-affected. The normal chest X-ray images (as shown in the left image) display clear lung fields without any signs of abnormal opacities. The image indicative of bacterial pneumonia (as seen in the middle image) typically displays focal lobar consolidation, evident in the right upper lobe (as indicated by the white arrow). Contrarily, viral pneumonia (right image) manifests a more diffuse “interstitial” pattern, involving both lungs.

By carefully curating and annotating this new dataset, we aim to enhance the accuracy and robustness of our AI system’s performance for pneumonia classification using chest X-ray images.

## Methods

In this study, we utilize a dataset comprising of 1,583 normal (non-pneumonia) images and 4,273 pneumonia images for pneumonia detection. The ensuing discussion will delve into the dataset’s specifics, the deep learning techniques adopted, and the models employed. Initially, an enhanced model was deployed for pneumonia classification, followed by the application of a CNN architecture to yield obtain the classification results. A comprehensive examination of these results is presented in the Experiments and results section, set within a Python environment.

### ResNet-50

**Fig. 2:**
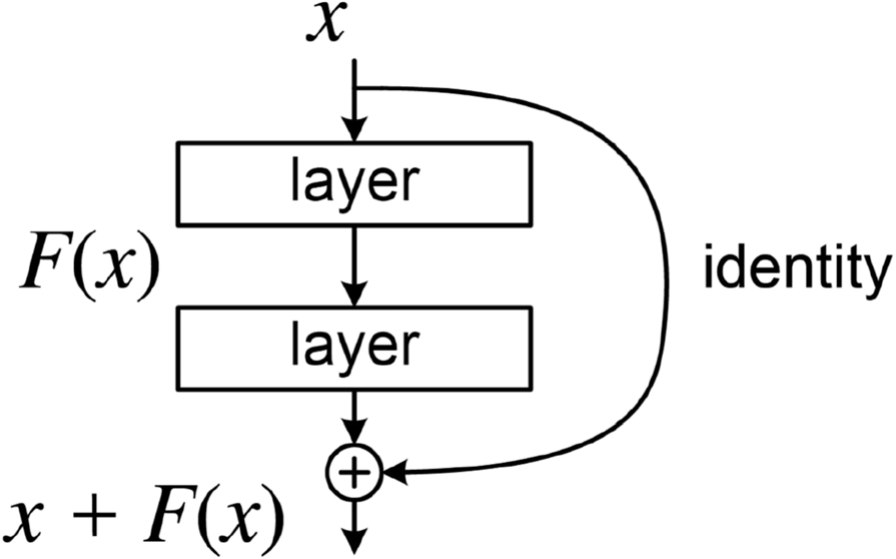
Resnet network architecture

The architecture of the ResNet-50 network is presented as follows,as show in Fig. 2. An example input image, colored and sized 224$\times$224 as an example, it first traverses a convolutional layer (conv1), which houses 64 filters, a kernel size of 7$\times$7, and a stride of 2. This layer yields images of dimensions 112$\times$112 with 64 channels. Subsequently, the image is directed through a 3$\times$3 max-pooling layer, resulting in images of dimensions 56$\times$56 and retaining 64 channels. Following this, four sequential residual blocks (namely *conv*2*x*, *conv*3*x*, *conv*4*x*, and *conv*5*x*) are arranged, and by which point, the output images possess dimensions of 7$\times$7 and encompass 2048 channels. Finally, an average pooling layer, a fully connected layer, and softmax activation are employed to calculate the classification probabilities.

### Attention

To discern more nuanced differences between pneumonia patients and healthy individuals, we propose a novel attention mechanism. This mechanism enhances feature extraction and promotes feature interactions by introducing side branches during the convolution process. These side branches, comprised of two convolutional kernels,generate two additional feature maps. Initially, the feature maps from the main and side branches undergo convolutional operations to reduce feature dimensionality, simplifying computational complexity while enabling the extraction of more representative features. Subsequently, these dimensionality-reduced feature maps are upscaled to their original dimensions using deconvolutional operations. Finally, one of the convolutional kernels is connected to the aggregate of the original feature map and the upsampled feature map from the side branches, resulting in the final feature map.

**Fig. 3:**
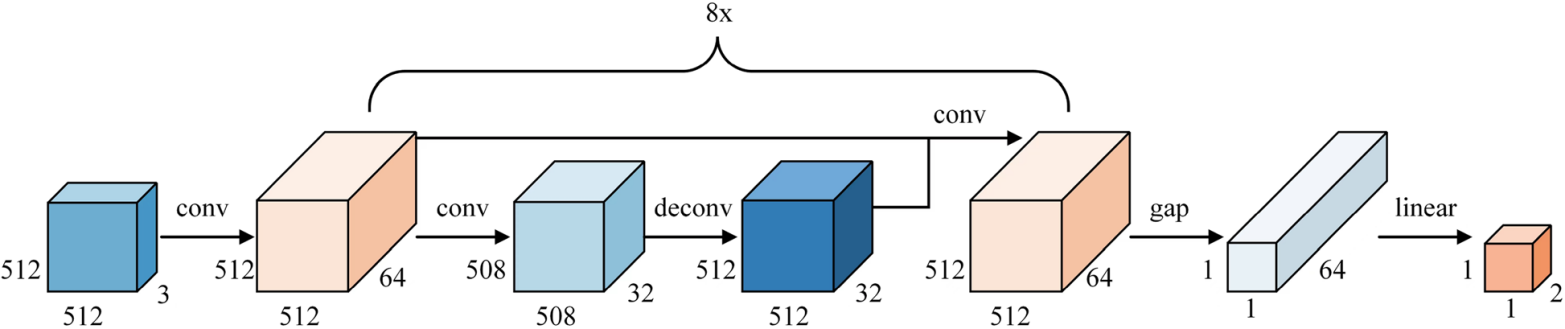
ResNet with additional skip connections

This attention mechanism bolsters the performance of the neural network by adaptively learning the correlation between different features and their importance. Figure 3 illustrates the complete architecture of our model. In this section, we will describe the constituents of our model in a step-by-step manner.

1. Input original image: Our original input image size was (512, 512, 3), which was first dimensionalized through a convolutional layer, and the channel was increased to 64.
2. Generation of Side Branches: The attention mechanism introduces two additional feature maps generated by applying additional convolutional kernels to the original feature map via side branches.
3. Convolutional Dimensionality Reduction: Convolutional operations are employed on the feature maps from the side branches to reduce their dimensionality. This strategy not only lowers computational complexity but also helps to extract more representative features.
4. Deconvolutional Upsampling: Subsequently, deconvolutional operations utilized to restore the original dimensions of the dimensionality-reduced feature maps.
5. Feature Map Fusion: The upsampled feature maps are fused with the original feature map via an element-wise addition. This fused feature map combines information from both the original feature map and the side branch convolutional feature maps.
6. Final Feature Map: We obtained the final feature map through 8 consecutive blocks, with a shape of (512, 512, 64). After passing through a gap (global mean pooling), we obtained (1, 1, 64), and then linearly transformed it to (1,1,2)for pneumonia binary classification.

By employing the steps above, the attention mechanism integrates additional side branches and amalgamates various feature maps. As a result, this leads to a more enriched feature representation and fostering feature interactions. These enhancements significantly improve the performance of the neural network in classifying pneumonia images.

The benefits and innovations of this approach include:

1. Enriched Feature Representation: By utilizing two dimensionality-reduced feature maps via the side branches, the model introduces a richer set of feature representations, which enhances its capability to effectively capture input features.
2. Feature Interaction: The fusion of the original feature map with the side branch convolutional feature maps enables interaction of features at different levels. This fosters improved information flow and reciprocal influence among features, thereby bolstering the model’s representation capacity.
3. Multi-scale Information Fusion: With convolutional dimensionality reduction and deconvolutional upsampling, the model can encode and decode features at varing scales. This capability aids in capturing multi-scale feature information, thereby enhancing object perception across different scales.
4. Gradient Propagation and Optimization: By introducing side branches, gradient propagation is enhanced, mitigating the vanishing gradient problem. This benefits the training and optimization of the network by ensuring stable and efficient gradient updates.

In summary, the innovation of this attention mechanism is characterized by the introduction of side branches and the strategic design of multi-layer feature interactions. These modifications amplify the model’s capacity for feature representation and interaction. They effectively addresses the problems associated with inadequate feature representation and inefficient information transfer. This methodology offers a powerful means for fusing multi-scale feature information, thereby enhancing the model’s performance and its ability to generalize.

### Focal loss

Focal Loss [11], a loss function specifically engineered to mitigate the issue of class imbalance, is particularly effective in tasks related to object detection. This method was introduced by Lin et al. in their 2017 paper, “Focal Loss for Dense Object Detection”. In the context of object detection tasks, class imbalance refers to a situation in which the count of negative samples (background) substantially surpasses the number of positive samples (objects of interest). Traditional cross-entropy loss functions often underperform when dealing with class-imbalanced data, as they are inherently biased towards the majority class (negative samples). This bias consequently leads to decreased predictive performance for the minority class (positive samples).

Focal Loss addresses this imbalance by integrating a modulating factor that adjusts the impact of each training sample to the total loss based on its predicted probability. The magnitude of this modulating factor is directly contingent on the predicted probability value: a higher predicted probability results in a smaller modulating factor, whereas a lower predicted probability leads to a larger one.

The mathematical definition of Focal Loss can be expressed as follows:

$$\begin{aligned} FL(p_t) = -\alpha_t (1-p_t)^{\gamma}{log{(p_t)}}\end{aligned}$$

The key components of Focal Loss include:

$FL(p_t)$: Denotes the Focal Loss for an individual training sample. $p_t$: Signifies the predicted probability of the actual class for a given sample. $\alpha _t$: This is the modulating factor that adjusts the weight of the loss for each sample according to its class. It aids in balancing the influence of easy and difficult samples. $\gamma$: This parameter that controls the rate at which the modulating factor decreases with increasing predicted probability.

By incorporating Focal Loss, the model can allocate more attention to samples that are difficult to classify, thereby diminishing the influence of simpler samples. This, in turn, enhances the model’s performance on minority classes. The parameters $\alpha _t$ and $\gamma$ of Focal Loss can be adjusted according to the specific requirements of the task. Generally, a larger $\gamma$ value can give more emphasis to hard samples, while a smaller $\gamma$ value strikes a balance between simpler and more challenging samples. Tuning these parameters may require iterative experimentation to identify optimal values.

To sum up, Focal Loss is a loss function specifically designed to address class imbalance by employing modulating factors to adjust the weights of training samples. This allows the model to concentrate more effectively on challenging samples and subsequently enhances its performance on minority classes in tasks related to object detection.

Despite the significant success of Focal Loss in addressing class imbalance problems, there are several associated limitations and drawbacks, which include:

1. Hyperparameter Selection: Focal Loss relies on two key hyperparameters, $\gamma$ for the modulating factor and $\alpha$ for the weight factor. Selecting appropriate values for $\gamma$ and $\alpha$ is crucial for obtaining optimal performance, but it requires experimentation and empirical tuning. Different datasets and tasks might require distinct hyperparameter settings, necessitating hyperparameter optimization when using Focal Loss.
2. Sensitivity: Focal Loss is highly sensitive to the choice of $\gamma$ and $\alpha$. Small adjustments to these hyperparameters can have a significant impact on the final performance. This implies that applying Focal Loss demands more time and effort for hyperparameter tuning and model optimization.
3. Dependency on Sample Distribution: Therefore, the application of Focal Loss necessitates by the distribution of samples. When training data exhibit extreme class imbalance, Focal Loss might not fully address the issue. Additionally, changes in class distribution within the training data can also influence Focal Loss’s performance.
4. Applicability to Other Tasks: While Focal Loss has been successful in object detection tasks, it may not be suitable for other types of tasks, such as semantic segmentation or instance segmentation. Different tasks have distinct features and data distributions, requiring specific loss functions and techniques to address class imbalance issues.

In conclusion, although Focal Loss has certain advantages in tackling class imbalance problems, it still has some drawbacks and limitations. The use of Focal Loss necessitates careful hyperparameter tuning, and the specific characteristics of the task should be taken into account. Additionally, for different tasks and datasets, further exploration and the development of new methods may be required to effectively mitigate class imbalance issues.

For this purpose, we have made improvements based on the Focal Loss to more effectively address the issue of class imbalance. The specific formula is as follows:

$$\begin{aligned} Loss = \left\{ \begin{array}{ll} \frac{-1}{\sqrt{\text {num\_sample\_normal}}}*\alpha *(1-y^{'})^{\gamma }\log {y^{'}} &{} y = 1 \\ \frac{-1}{\sqrt{\text {num\_sample\_penumonia}}}*(1-\alpha )*(y^{'})^{\gamma }\log {(1-y^{'})} &{} y = 0 \\ \end{array}\right. \end{aligned}$$

By introducing these additional adjustments to the original Focal Loss, the modified loss aims to improve the model’s ability to handle class imbalance, facilitate the learning process, and boost the performance of the model on imbalanced datasets.

## Experiments and results

In this section, we describe several experiments conducted to propose a more effective model for the detection of pneumonia. The objective of these experiments is to explore various approaches and configurations to find an optimal model for pneumonia detection. Through these experiments, we aim to identify the most suitable algorithms and parameter settings that can achieve the best performance and accuracy in pneumonia detection.

### Dataset split

We meticulously collected and annotated a total of 5,856 chest X-ray images of children, including 4,273 images depict pneumonia, and 1,583 images are normal. The dataset was carefully curated to guarantee its quality and accuracy.

For training our model, we used a dataset from 5,440 patients, comprising 4,160 images of pneumonia cases and 1,280 images representing normal cases. This large and diverse training dataset allowed the model to learn and generalize from a wide range of cases.

To evaluate the performance of our AI system, we created a validation set consisting of 208 normal chest X-ray images and 208 images showing pneumonia. Distinct from the training dataset, these images were collected from a separate set of 416 patients. The validation set was used to assess the model’s ability to generalize to new and unseen cases.

The dataset additional descriptions are as follows:

1. Contrast: Contrast = 0.9982. Contrast refers to the measurement of different brightness levels between the brightest white and darkest black areas in an image. A larger difference range represents higher contrast, while a smaller difference range indicates lower contrast.
2. Spatial Resolution: Spatial Resolution = 132.89. It refers to the size or dimension of the smallest unit on an image that can be used to differentiate details. It is an indicator used to characterize the level of detail that can be resolved in ground targets in the image.
3. Noise: Noise = 6.6923. This means unnecessary or unwanted interference information present in the image data.

### Feature extractor

For image feature extraction, this study utilized the pretrained model ResNet-50 as a baseline to evaluate its performance in classifying abnormal and normal chest X-ray images. On this basis, we introduced the use of skip connections and an improved focal loss. Table 1 presents the performance of the baseline model and the improved model in classifying abnormal and normal chest X-ray images.

The results showed that the ResNet-50 with skip connections and focal loss outperformed the pretrained ResNet-50 model, achieving an accuracy score of 0.98.

### Evaluation metrics

**Fig. 4:**
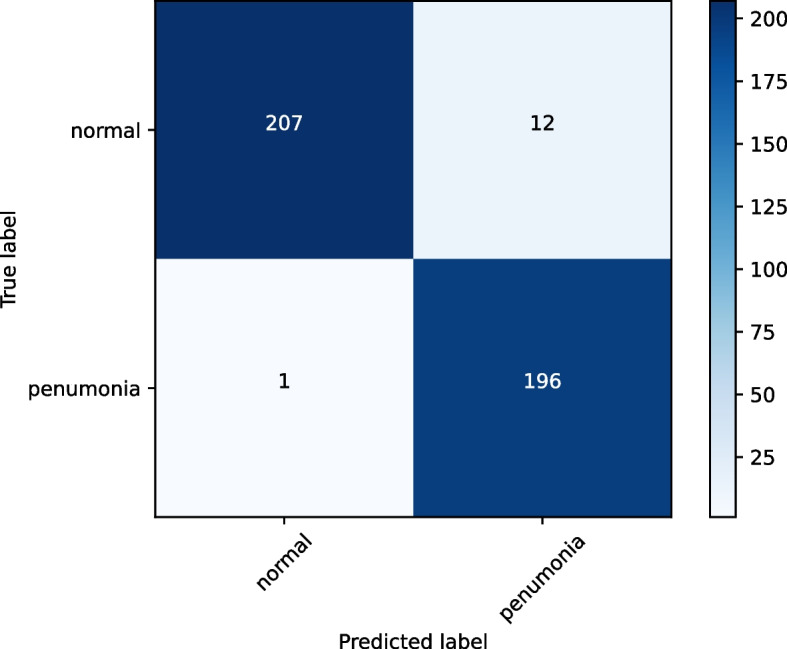
Confusion matrix

The confusion matrix is used to describe the performance of a classification model and provides a visual representation of its performance. The confusion matrix is typically presented as shown in Fig. 4:

In this experiment, we evaluated the model’s performance using a confusion matrix. The values of TP=207, FP=12, TN=196, and FN=1 were obtained. High TP and TN values indicate accurate predictions, but the presence of FP and FN values suggests misclassifications. Overall, the model showed promising performance.

Accuracy, a key metric in model evaluation,is defined as the ratio of the number of correctly predicted instances to the total number of predictions. It can be calculated using the following formula:

$$\begin{aligned} Accuracy = \frac{\text {Number of Correct Predictions}}{\text {Total Number of Predictions}} \end{aligned}$$

Specificity is a metric related to the classifier’s ability to detect negative results [12]. It can be calculated using the following formula:

$$\begin{aligned} Specificity = \frac{\text {True Negative}}{\text {True Negative + False Positive}} \end{aligned}$$

Precision is the ratio of true positive predictions to the total predicted positive instances [13]. It can be calculated using the following formula:

$$\begin{aligned} Precision = \frac{\text {True Positive}}{\text {True Positive + False Positive}} \end{aligned}$$

Recall, also known as sensitivity or true positive rate, represents the ratio of correctly classified positive instances to the total number of positive instances. It can be calculated using the following formula:

$$\begin{aligned} Recall = \frac{\text {True Positive}}{\text {True Positive + False Negative}} \end{aligned}$$

### Training configuration

**Fig. 5:**
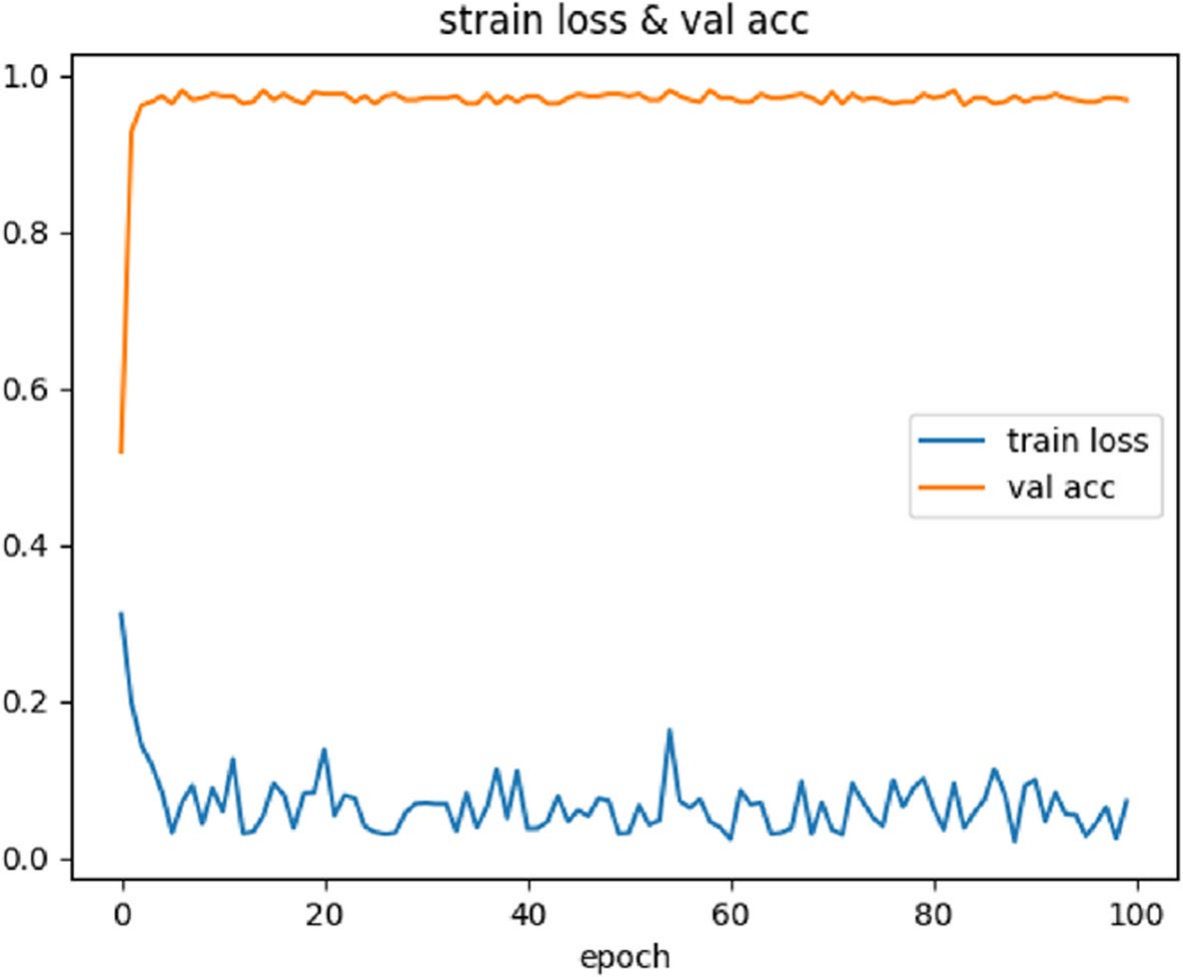
Training loss and Validation accuracy

The training of our model was performed using the PyTorch framework. The batch size for the experiments was set to 16. We used the AdamW optimizer with an initial learning rate of 0.00005, a momentum of 0.9 for parameter updates, and a weight decay coefficient of 0.0001. The StepLR scheduler was employed, which reduced the learning rate by a factor of 0.1 after every 5 epochs. The training was stopped after 100 epochs (100 iterations over the entire dataset) as shown in Fig. 5.

During the training process, the validation accuracy gradually improves with the increasing number of epochs. In the first 8 epochs, the accuracy consistently rises, while the loss continuously decreases. These observations indicate that the model is making significant progress in learning data features and enhancing its generalization capabilities.

### Results

**Fig. 6:**
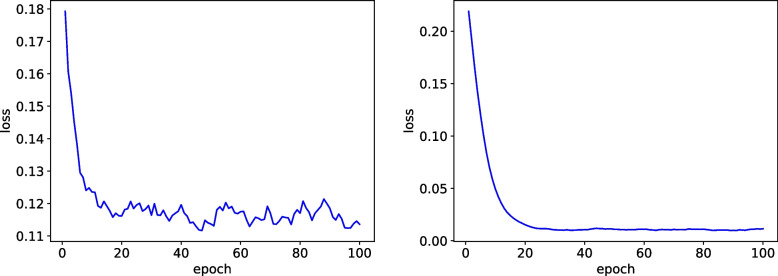
Left: Training loss, Right: Val loss

In this investigation, a series of experiments were conducted to assess the performance of various deep learning models in detecting pneumonia from chest X-ray images. The baseline model, ResNet-50, achieved a satisfactory accuracy of 0.94,as show in Fig. 6, demonstrating its effectiveness in this task. However, to further enhance the model’s performance, we incorporated additional components.

**Fig. 7:**
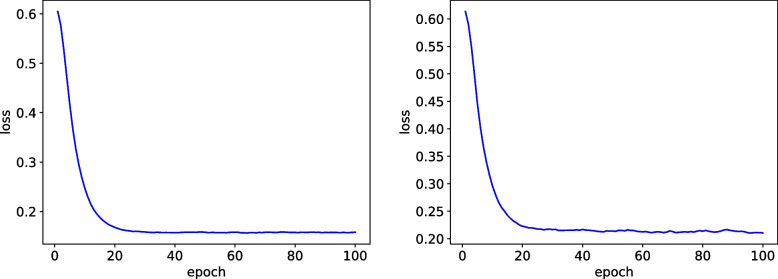
Left: Training loss, Right: Val loss

In the first enhancement, we incorporated skip connections into the ResNet-50 architecture. Skip connections effectively captured more informative features and facilitated feature interaction. This led to improved information propagation and feature representation. As a result, the ResNet-50 model with skip connections demonstrated a significant accuracy of 0.96,as show in Fig. 7, indicating a substantial improvement over the baseline.

**Fig. 8:**
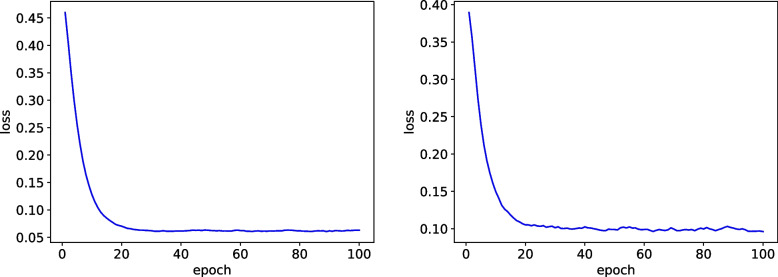
Left: Training loss, Right: Val loss

Encouraged by this progress, we explored a further refinement by integrating the Focal Loss function into the ResNet-50 model with skip connections. Focal Loss has demonstrated efficacy in addressing class imbalance, which is a significant issue in datasets where pneumonia samples vastly outnumber normal samples. Through adaptive weighting of the loss for individual samples based on their prediction probabilities, Focal Loss prioritized challenging samples, thus enhancing the model’s ability to detect pneumonia. Remarkably, this enhanced model achieved an impressive accuracy of 0.98,as show in Fig. 8, surpassing both the baseline and the model with skip connections. After the above improvements in our experiment, on the one hand, we added a skip connections in the completion process, and also improved the FocalLoss loss function. In the end, our results improved by 0.04, which is a great improvement.

### Evaluation

**Fig. 9:**
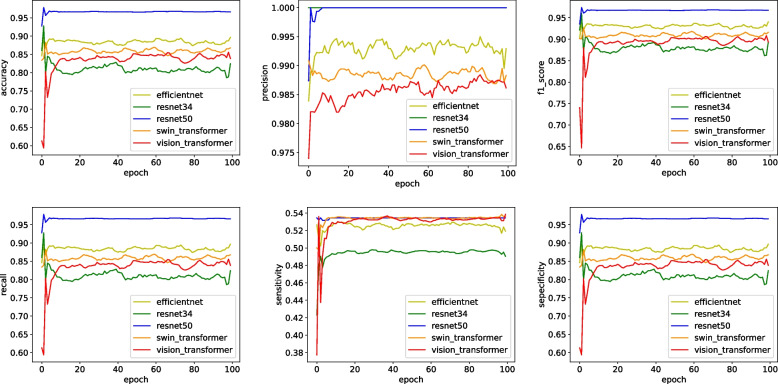
Comparative experiment

In order to further highlight the effect of our model,we compare the proposed method with four methods: Efficient-b2 [14], Resnet34 [8], Swin transformer [15], and Vision transformer [16], as show in Fig. 9.

The results of these experiments underscore the importance of thoughtful design and optimization of deep learning models for medical image analysis tasks,as show in Table 1. The incorporation of skip connections and Focal Loss has exhibited substantial advantages in capturing more informative features and addressing class imbalance issues, respectively. The superior accuracy of the improved model in pneumonia detection underscores its potential in assisting medical professionals with accurate diagnoses of this critical condition.

**Table 1:** Ablation experiment

Method	Accuracy
ResNet-50	0.94
ResNet-50 + skip connetions	0.96
ResNet-50 + skip connetions + Focal loss	0.98

The improved ResNet50 outperforms other models in terms of accuracy, F1 score, recall, sensitivity, and specificity,as show in Table 2. It only slightly lags behind ResNet34 in precision.

**Table 2:** Performance Metrics for Different Models

Model	Accuracy	F1 Score	Precision	Recall	Sensitivity	Specificity
Efficient-b2	0.8872	0.9372	0.9932	0.8872	0.5264	0.8872
Resnet34	0.8944	0.9442	1	0.8944	0.495	0.8944
**our improved Resnet50**	0.9868	0.9825	0.9989	0.9668	0.5344	0.9668
Swin Transformer	0.8591	0.9173	0.9908	0.8541	0.5341	0.8591
Vision Transformer	0.8373	0.9055	0.9859	0.8373	0.5311	0.8373

In conclusion, our study emphasizes the significance of synergizing advanced deep learning architectures and loss functions to achieve remarkable performance in medical image analysis. The findings highlight the potential of such approaches in enhancing diagnostic accuracy and facilitating clinical decision-making. As medical imaging technologies continue to advance, these models hold promise in aiding healthcare practitioners with precise pneumonia diagnoses and potentially other diseases, ultimately leading to improved patient outcomes and healthcare quality.

## Discussion

Through customizing the model, specifically by combining CNN-based feature extraction and supervised classification algorithms, we achieved an optimal solution for classifying abnormal (labeled as pneumonia) and normal chest X-ray image. This success can be attributed primarily to the advanced features provided by ResNets, which represent a significant breakthrough in deep neural networks. In ResNet, skip connections are deployed to mitigate the vanishing gradient problem, a common issue encountered in deep learning. To improve the accuracy and precision of both training and testing processes, we incorporated additional layers, including ... [Specify the type of layers] into the ResNet model. Despite these improvements, in our study, we observed that training a neural network with a very large number of layers using lung CT scan data still encounters the vanishing gradient problem. This problem, which typically arises with deeper networks, can lead to a degradation in model performance.

## Conclusion

In recent years, the application of deep learning methods in clinical and radiology settings has rapidly increased. This study aimed to utilize deep learning architectures for detecting pneumonia in X-ray images. Utilizing the foundation of the ResNet-50 model, we introduced new image processing branches, and made specific enhancements to the ResNet-50 model. In conjunction with these modifications, we proposed and implemented a new loss function to optimize the model. Ultimately, the improved model achieved the highest accuracy. This sophisticated model for detecting pneumonia in patient images will aid medical experts in making more precise inferences about the disease. Furthermore, it holds potential to mitigate diagnostic errors typically associated with traditional methods.

## Data Availability

The data used in this study is an open-source pneumonia classification dataset collected from online sources. The dataset can be accessed at https://blog.csdn.net/ispeasant/article/details/124228341 (or other means specified). The experiments conducted in this paper strictly adhere to the terms and conditions of the open-source license. Detailed information regarding data preprocessing and analysis methods can be found in the supplementary materials of the open-source dataset.
